# JNK Cascade-Induced Apoptosis—A Unique Role in GqPCR Signaling

**DOI:** 10.3390/ijms241713527

**Published:** 2023-08-31

**Authors:** Guy Nadel, Galia Maik-Rachline, Rony Seger

**Affiliations:** Department of Immunology and Regenerative Biology, Weizmann Institute of Science, Rehovot 7610001, Israel; guy.nadel@weizmann.ac.il (G.N.); galia.maik-rachline@weizmann.ac.il (G.M.-R.)

**Keywords:** MAPK, JNK, GqPCR, apoptosis, PP2A switch, AKT, PI3K

## Abstract

The response of cells to extracellular signals is mediated by a variety of intracellular signaling pathways that determine stimulus-dependent cell fates. One such pathway is the cJun-N-terminal Kinase (JNK) cascade, which is mainly involved in stress-related processes. The cascade transmits its signals via a sequential activation of protein kinases, organized into three to five tiers. Proper regulation is essential for securing a proper cell fate after stimulation, and the mechanisms that regulate this cascade may involve the following: (1) Activatory or inhibitory phosphorylations, which induce or abolish signal transmission. (2) Regulatory dephosphorylation by various phosphatases. (3) Scaffold proteins that bring distinct components of the cascade in close proximity to each other. (4) Dynamic change of subcellular localization of the cascade’s components. (5) Degradation of some of the components. In this review, we cover these regulatory mechanisms and emphasize the mechanism by which the JNK cascade transmits apoptotic signals. We also describe the newly discovered PP2A switch, which is an important mechanism for JNK activation that induces apoptosis downstream of the Gq protein coupled receptors. Since the JNK cascade is involved in many cellular processes that determine cell fate, addressing its regulatory mechanisms might reveal new ways to treat JNK-dependent pathologies.

## 1. Introduction

The response of cells to extracellular signals is mediated by a variety of intracellular signaling pathways that are responsible for the stimulus-dependent cell fate [1]. A group of signaling pathways are the mitogen-activated protein kinase (MAPK) signaling cascades that are involved in most stimulated cellular processes [2,3,4,5]. Four MAPK cascades have been identified in mammals over the past 35 years, providing evolutionary-conserved mechanisms of signal transduction. They are usually termed by the components at the MAPK tier: ERK1/2 (ERK; [6,7]), JNK [8,9], p38 [10,11], and ERK5 [12], that cumulatively transmit signals of essentially all extracellular stimuli, including hormones, growth factors, and environmental changes. The cascades transmit their signals via a sequential activation of protein kinases organized as a core of three tiers (MAP3K, MAPKK, and MAPK), and in some cases contain an upstream MAP4K and downstream MAPKAPK tiers. The strength and duration of the signals transmitted via each cascade vary between stimulants and cells, as the ERK1 cascade is mainly linked to proliferation and survival, while the p38/JNK cascade usually mediates stress responses, and ERK5 may be involved in both processes. In this review, we cover several aspects of the regulation of the JNK cascade and its role in extracellular signal-induced apoptosis. We also cover in detail the mechanism of JNK activation and induced apoptosis downstream of the Gq protein-coupled receptors (GqPCRs), which has not been reviewed thus far.

## 2. Components and Activation of the JNK Cascade

The JNK (c-Jun N-terminal kinase) cascade is initiated by various extracellular and intracellular stimuli that are usually stress-related but may also induce some stress-independent processes. The signals of extracellular stress factors are mainly transmitted by stress/apoptosis-related receptors (e.g., TNF receptor [13]) at the plasma membrane, while stress-related physical changes (e.g., osmotic pressure [14]) can transmit their signals by either physical changes or receptor activation at the plasma membrane [15]. On the other hand, most of the stress-independent JNK-activating factors (e.g., EGF [16]) operate via GPCRs and RTKs, and the internal stresses (e.g., ER stress [17]) are mediated by unique and dedicated signaling pathways [8]. Upon stimulation, the various receptors or internal stresses further transmit the signals to the MAP3K of the JNK cascade via two principal types of mechanisms. One of them is stimulation-induced changes in dedicated scaffold proteins that in turn recruit and activate protein kinases at the MAP4K of the JNK cascade (e.g., TRAF2 that recruits and activates the MAP4K GCK1 [18]) to further activate MAP3K components [19]. However, in some cases, the adaptor proteins can directly activate MAP3Ks (e.g., MEKK1-5 [20]), skipping the MAP4K level [21]. The other mechanism involves receptor-mediated activation of small GTPases (e.g., Rac, Rho, and CDC42 [22]) that directly activate MAP3Ks in the cascade [23]. There are about 17 protein kinases that can act as MAP3K in the JNK cascade [2], and once one or more of them is activated, they further transmit the signals to the MAPKK tier, which includes MKK4 and MKK7. The mechanism of stimulated MAP3K activation may be regulated not only by activatory phosphorylation or autophosphorylation but also by a relief from autoinhibition seen in some of them (e.g., MEKK1 [24]) and dimerization that enhances their activity (e.g., MEKK2 [25]). The two MAPKKs can further phosphorylate the Tyr and Thr residues that lie within the TPY motif in the activation loop of JNKs and thereby activate them [8]. Although each of the MAPKKs can act by itself to induce full activation of JNKs, it was shown that MKK4 has a preference for the tyrosine residue, while MKK7 prefers the Thr residue, inducing a synergistic activation by both MAPKKs [26]. Three genes (MAPK8/JNK1, MAPK9/JNK2, and MAPK10/JNK3) encode for the proteins in the MAPK tier of the cascade, which are translated into not less than eleven alternatively spliced isoforms, all with a similar activity [5]. JNK1 and JNK2 have eight spliced isoforms, all with MW of either 46 or 54 kDa, that are ubiquitous and abundant, while the three alternatively spliced isoforms of JNK3 (50, 54, and 58 kDa) are tissue-specific. These isoforms seem to have very similar activities in most cell lines and tissues. Finally, unlike ERK and p38, very little is known about JNK’s MAPKAPKs, as the only known one is 3pK (MAPKAPK3) and its function within the cascade is not fully understood yet [27].

## 3. Downregulation of JNK Activity 

**Figure 1:**
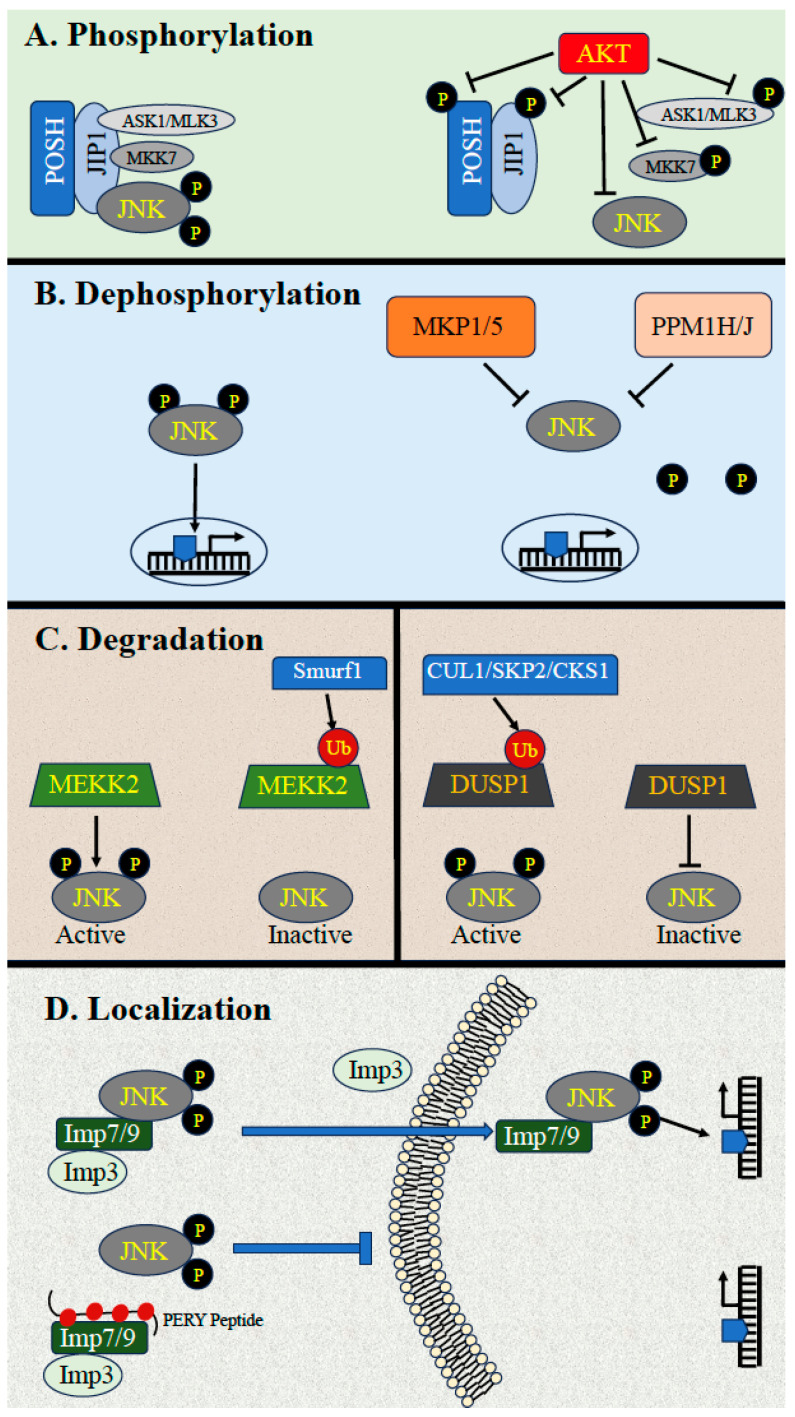
Downregulation of JNK activity. Different mechanisms that downregulate the JNK cascade. (**A**)**.** Downregulation by phosphorylation. The JNK cascade’s components are attached to the scaffold proteins POSH and JIP1-4 under stress conditions (left side). This is downregulated (right side) when AKT phosphorylates the scaffolds or cascade’s components to block JNK activation. (P—phosphate) (**B**). Dephosphorylation. Upon stimulation, JNK is phoshorylated and activated to further phosphorylate its nuclear substrates. Protein phosphatases (e.g., MKP1/5, PPM1H) remove these phosphates and inactivate JNK to block transcription. (**C**). Degradation. The JNK cascade is inhibited by Ubiquitin mediated degradation of some of its components. MEKK2 is ubiquitinated (Ub) in a Smurf1 dependent manner (left panel), to inhibit JNK phosphorylation and activation. Alternatively, CUL1/SKP2/CKS1-dependent ubiquitination can induce JNK activation when it causes degradation of phosphatase DUSP1 (MKP1). (**D**). Localization. Activated JNK is translocated into the nucleus upon stimulation by beta-like importins (Imp7, Imp9 and Imp3; upper panel). This can be inhibited by either irreversible interaction of JNK with anchoring proteins or by an inhibitory peptide (PERY) that prevents nuclear JNK translocation and thereby can be used for therapeutic purposes (lower panel). More information is found in the text.

Apart from the sequential phosphorylation of the JNK cascade’s components, the transmission of signals via this pathway is stringently regulated by several other processes, including phosphorylation, dephosphorylation, degradation, subcellular localization, and protein-protein interactions [7,28,29,30,31,32]. These mechanisms are responsible for determining the specificity of the cascade by dictating the duration, strength, and substrate specificity of the signals. As mentioned above, the activation is regulated mainly by activating phosphorylation, which is often modulated by other intracellular components. These include scaffold proteins and other regulators that bring the components into close proximity to their proper activators and effectors, thus enhancing the signaling kinetics and determining proper localization. These activatory parameters have already been well covered in previous reviews [8,21,33,34]. On the other hand, the mode of inactivation or downregulation of the cascade, which is mainly mediated by phosphorylation/dephosphorylation and supported by degradation, protein interaction, and changes in localization, is not covered as well. These points are described below (see also Figure 1).

### 3.1. Downregulation by Phosphorylation

A prominent mechanism by which the JNK cascade is negatively regulated is the phosphorylation of components of the JNK cascade by the survival protein kinase AKT. This mode of regulation is mainly important to prevent the apoptotic effects of JNK and thus secure cell survival. In this context, it has been shown that some of the MAP3Ks and MAPKKs in the JNK cascade are phosphorylated by AKT, which inhibits their activation in response to extracellular stimulation, particularly stress. This includes the inhibitory phosphorylation of Ser83 of the MAP3K ASK1 [35], Ser674 of the MAP3K MLK3 [30,36,37], and Ser80 of the MAPKK MKK4 [38]. Apart from the inhibitory phosphorylations of the kinase components of the cascade, AKT may regulate the JNK cascade by phosphorylating the scaffold of the cascade. Indeed, it was shown that the JNK scaffold JIP1 [39] interacts with AKT, thus perturbing the interaction between JIP1 and JNK cascade kinases, leading to reduced JNK signaling in neurons [39]. A similar effect was seen with the scaffold protein POSH, whose binding to AKT2 upon stimulation reduces the interaction with components of the cascade and thereby reduces activity [40]. In addition, the JNK cascade can be regulated by AKT through phosphorylation of Ser71 of the small GTPase Rac1 [41], leading to its inactivation, which consequently inhibits the JNK cascade and provides a protective mechanism against ischemic brain injury. Interestingly, the activity of the JNK cascade may also be downregulated by GSK3β, which is often regulated downstream of AKT. Thus, GSK3β was shown to interact with and phosphorylate the MAP3K MEKK4, to inhibit its activity and reduce JNK activation [42].

### 3.2. Downregulation by Dephosphorylation

Another important mechanism of JNK downregulation is the dephosphorylation of the components of the JNK cascade by various protein phosphatases. As the protein kinases in this pathway are activated by phosphorylation, the removal of the activating phosphates results in the inactivation of the cascade’s kinases. The upstream components (MAP3Ks, MAP2Ks) are all activated by the incorporation of phosphates into activatory Ser/Thr residues. Therefore, the activity of these components is downregulated by Ser/Thr phosphatases such as PP2A, PP5, and others that are not specific to the JNK cascade. These Ser/Thr phosphatases may also regulate JNK itself, dephosphorylating the activatory pThr (but not pTyr) and reducing JNK activity. Such an inactivation might also be mediated by the proteins Ser/Thr metallophosphatase, PPM1J [43], and PPM1H [8], which harbor a JNK-binding motif, indicating that they may either dephosphorylate the activatory pThr of JNK or possibly dephosphorylate upstream components that are recruited to JNK via scaffold proteins. Interestingly, the possibility of protein Tyr phosphatase to inactivate JNK by specifically removing the phosphate from the activatory Tyr was also suggested [44]. This suggests that, in similarity to ERK [45], removal of the phosphates from both activatory pThr and pTyr residues can occur by two distinct Tyr and Ser/Thr phosphatases working together. However, a more common mode of JNK inactivation is mediated by MAPK phosphatases (MKPs), which are dual-specificity phosphatases (DUSP, [46]) that can each dephosphorylate both activatory residues of JNK [47]. Up to nine distinct DUSPs can directly regulate JNK [48], as briefly described below.

### 3.3. Dephosphorylation by MKPs

The MKPs are a group of twelve phosphatases (ten typical and two atypical) that simultaneously dephosphorylate the two activatory pTyr and pThr residues, which leads to a complete inactivation of the MAPKs. The only MKP that was suggested to be quite specific to JNK is MKP7 (DUSP16; [49]), and indeed, it was shown that it down-regulates JNK-dependent processes in some systems (e.g., [50]). However, it should be noted that recent studies have demonstrated that MKP7 can also dephosphorylate p38 [51] and even ERK [52]. Therefore, more studies are required to clarify the specificity of this MKP. In addition, JNK can be dephosphorylated by other MKPs [48], with a preference to p38/JNK over ERK (MKP1 and MKP5), preference to ERK/JNK over p38 (MKP2), preference to ERK over p38/JNK (MKP4), and by MKPs that dephosphorylate equally all MAPKs (PAC1, MKPX, hVH5, and MKP6). Interestingly, the specificity of the MKPs to ERK or p38 is mediated by a region in the phosphatases termed the rhodanese domain that interacts with the common docking motif of the kinases [53,54]. However, JNK cannot bind the rhodanese domains but rather interacts with other sequences, as observed in MKP5 and MKP7 [55,56]. Finally, MKPs may be under transcriptional or stability controls by their cognate MAPK cascades [46]. Such regulation was shown in drosophila, in which the MKP5 ortholog (Puckered) is upregulated by the activity of its JNK ortholog (Basket; [57]), and in mammals, JNK may partially control the expression of its downstream transcription factors [58]. This type of regulation can result in negative, or sometimes even positive, feedback loops in the cascade. Thus, the MKPs are an important group of JNK regulators that play a role in the regulation of most JNK-dependent cellular processes. 

### 3.4. Downregulation by Scaffold Proteins and Ubiquitin-Dependent Degradation

An additional mechanism for the downregulation of JNK is its interaction with anchoring or scaffold proteins [59]. The anchoring proteins interact with one of the components of the cascade and are responsible mainly for its proper subcellular localization. Scaffolds are defined as proteins that interact with more than one component of the cascade, usually leading to faster signaling, better interaction with certain upstream or downstream proteins, and proper localization. In most cases, these interacting proteins result in faster signaling kinetics but can also be involved in a negative regulation, mainly by recruiting phosphatases to JNK. An example of such an inactivation was reported for JIP3, which induces the interaction of the phosphatase M3/6 (DUSP8) with a complex of JNK cascade components, leading to reduced signaling [60]. Similarly, beta-arrestin2 was shown to recruit MKP7 in close vicinity to JNK3, leading to its inactivation [61]. It is likely that other scaffolds may be involved in downregulating the cascade through ubiquitin-mediated degradation [62,63]. Indeed, this type of degradation, which might also be mediated without scaffolding interactions, seems to be an important regulator of the JNK cascade during various processes [64]. Moreover, downregulation by degradation was reported for MKK4, which undergoes stimulation-dependent ubiquitination and degradation [65]. MKK4 ubiquitination requires JNK-dependent activation of the ubiquitin protein ligase Itch that interacts with the MAPKK, forming a negative feedback loop within the cascade. Other components that are downregulated by degradation are MAP3K, DLK1, and MEKK2, which are ubiquitinated and inactivated. Thus, DLK3 and MEKK2 are regulated by the ubiquitin ligases RPM1 and Smurf1, respectively, leading to their degradation [66,67,68]. Finally, the cascade’s regulation might involve ubiquitination of DUSPs [69] and downstream targets (e.g., Jun [70]), showing again the importance of degradation in downregulating the JNK cascade. 

### 3.5. Downregulation through Dynamic Subcellular Localizations

An additional mode of JNK regulation that can lead either to activation or downregulation of the cascade is the dynamic subcellular localization of its components. In resting cells, JNK and the other components are localized primarily in the cell’s cytoplasm or in some cytoplasmic organelles [8]. Such distinct localizations can participate in the determination of signaling specificity upon distinct stimulations. An example of such regulating distribution in resting cells is JIP3-mediated JNK localization in exocytic vesicles and focal adhesions to execute specific JNK activities [71]. Interaction with JAMP that localizes JNK to the plasma membrane may be involved in extending JNK signaling duration [72]. Finally, palmitoylation of JNK3 in nerve cells recruits it to the actin cytoskeleton to specifically regulate axonal development [73]. Importantly, the localization is changed upon extracellular stimulation, which induces not only JNK activation but also changes in the subcellular localization of some JNK cascade components. This process is also required to determine the proper function and specificity of the cascade [2]. In particular, stimulation of JNK induces its nuclear translocation to activate nuclear factors necessary for JNK-induced cell fate [31]. The mechanism of nuclear translocation of some JNK cascade components involves the interaction of Impα/β with their canonical NLS. However, JNK does not have an NLS, and its mechanism of translocation involves stimulated interaction with a dimer of beta-like importins (Imp3/7 or Imp3/9) that escort JNK to the nucleus to allow proper cellular processes [31,74]. It was also suggested that, in some cells, the first part of the translocation is mediated by kinesin1, which transports JNK on microtubules to reach the close vicinity of the nucleus [75]. Thus, JNK activity can be regulated by either the prevention or acceleration of its nuclear translocation. An example of such prevention is the MAP3K DLK, whose various activities are dependent on its distinct localizations [76]. Additionally, MKK7γ localization is regulated by its interaction with the phosphatase PP2B, which not only dephosphorylates it but also keeps it in the cytoplasm [77]. Finally, when cytoplasmic anchoring of JNK1 by MKK7 is interrupted, the cells become resistant to Fas-mediated apoptosis [78]. Overall, JNK activity is well regulated by a variety of mechanisms to determine proper specificity and cell fate. 

## 4. Cellular Processes Regulated by the JNK Cascade

The JNK cascade is involved mostly in the regulation of stress-related processes, although it can participate in the regulation of many other processes as well. This is a common feature of all MAPKs that may participate in the regulation of distinct and even opposing processes dependent on the cellular context and distinct cellular wiring [8,9]. Over the years, it has been found that the JNK cascade may be involved in processes like apoptosis [14,79], unfolded protein response (UPR; [9], neuronal functions [80], embryonic development [81], metabolism [82], longevity [83], immunity [84], and others. As a central regulator of many cellular processes, dysregulation of the cascade leads to various diseases, including neurodegenerative diseases [85], insulin resistance and metabolic disorders [86], inflammation [87], some cancers [88], and more. Here we describe the role of the JNK cascade in apoptosis, which is an important step in the induction of some of the physiological and pathological processes mentioned above. 

### 4.1. Role of the JNK Cascade in Apoptosis 

#### 4.1.1. A Brief Review of Apoptosis

Apoptosis is a major type of regulated cell death that plays an essential role in various developmental processes as well as in response to environmental changes [89]. The mechanisms involved in the process are mainly divided into extrinsic or intrinsic pathways based on their upstream stimulators [90]. The intrinsic one is mediated by internal cellular pathways, while the extrinsic one is mainly induced and acutely activated by extracellular stimuli [91,92,93]. Crosstalk between these two mechanisms can occur as well, particularly by activating the extrinsic machinery by the intrinsic ones. Both these pathways eventually stimulate a set of proteinases termed executive caspases (e.g., caspases-3,6,7). These caspases are directly responsible for the degradation of proteins as well as the activation of DNases and lipid modifiers, which are all essential for cell death [94]. A brief description of the upstream mechanisms:

*The intrinsic pathway*—This pathway, also known as the mitochondrial pathway, is usually stimulated by various internal stresses such as ER stress, DNA damage, and reactive oxygen species (ROS) production. They operate via the activation of pro-apoptotic proteins from the Bcl2 family, such as BAX and BAK, that, in turn, lead to the permeabilization of outer mitochondrial membranes. This allows the release of pro-apoptotic proteins from the mitochondria, which further advances the apoptotic pathway. Among these proteins is cytochrome C, which is important to produce the cytoplasmic apoptosome complexes. This causes the cleavage and activation of caspase-9, which in turn activates execution caspases including caspase-3 and caspase-7 that are essential for apoptosis. In parallel, the mitochondria secrete the proapoptotic proteins Smac/Diablo and Omi/HtrA2 that interact and block the action of negative regulators of apoptosis, thus allowing the essential step of caspase activation [95,96]. 

*The extrinsic pathway*—This pathway is initiated by extracellular stimuli that bind and activate several death receptors (e.g., TRAIL, Fas). Activation of the death receptors causes the formation, activation, and recruitment to the receptor of the death-induced signaling complex (DISC), which is composed of FAS-associated via death domain (FADD) and procaspase-8 that further induces the activation of executing caspases essential for the process. Other death receptors (e.g., TNFR, TRAMP) form another type of signaling complex, composed of TRADD, TRAF, and RIPK1, which are all termed complex1 and further transmit apoptotic signals and eventually induce activation of executing caspases. Apart from these pathways, both types of apoptosis can sometimes be mediated by autophagy, although this process usually leads to survival [97,98]. 

#### 4.1.2. JNK in the Induction and Regulation of Apoptosis

Apart from the death-mediating components of both intrinsic and extrinsic pathways, the processes may be mediated or regulated by additional enzymes that are often cell-type-specific. One group of regulators is the MAPKs, particularly JNK, which was shown to be involved in several processes within the intrinsic as well as the extrinsic pathways. JNK may affect apoptosis either directly by phosphorylating and regulating components of the apoptotic machineries or indirectly by regulating the expression of proteins involved. In addition, it was proposed that all JNK isoforms can induce apoptosis, and in some cases, this is mediated particularly by nuclear JNKs, while cytoplasmic JNKs participate in other processes [99]. Interestingly, JNK can induce anti-apoptotic processes as well [100], but those are usually not as prevalent as their role in promoting cell death. Some of the apoptosis-promoting effects of JNK include: 

*Direct effects on the intrinsic pathway*—JNK is a key regulator of the intrinsic pathway by regulating several Bcl2-related proteins. Among others, JNK was shown to phosphorylate and regulate both pro-apoptotic effectors to induce their activity (e.g., Bad) and anti-apoptotic effectors (e.g., Bcl-2) to inhibit it. Thus, JNK can directly phosphorylate the survival effectors Bcl-2 [101,102], Mcl-1 [103], and Noxa [104] to repress their anti-apoptotic function. On the other hand, JNK directly phosphorylates the pro-apoptotic Bad [105], Bim [106], and Bax [107] to induce their apoptotic activities. In addition, the phosphorylation of the apoptotic Bim and Bmf by JNK releases them from sequestering proteins to their binding to Bcl-2, neutralizes the survival function of the latter, and therefore induces Bax-related apoptosis [108]. Similarly, phosphorylation of 14-3-3, which is a cytoplasmic anchor, induces Bax release and translocation to the mitochondria [109], as well as the release of the pro-apoptotic proteins Bad [110] to induce apoptosis. 

*Direct effects on the extrinsic pathway*—TNFα, which is one of the best-studied death inducers via the extrinsic pathway, is known to operate via the JNK pathway. The mechanism by which JNK mediates the TNFα effect involves caspase-8-independent cleavage of Bid, releasing jBid that translocates to the mitochondria and selectively promotes the release of Smac/DIABLO to induce apoptosis [111]. Interestingly, direct Bid [112] or Smac/DIABLO [113] phosphorylation by JNK can induce such effects by themselves or enhance the effects of other upstream regulators. In addition, it has been reported that JNK phosphorylates the E3 ubiquitin ligase Itch, which in turn promotes ubiquitination-mediated degradation of the caspase-8 inhibitor c-FLIP [114], thus inducing caspase 8-mediated apoptosis [14]. Another mechanism is the phosphorylation of Bid at Thr59 by activated JNK. Although this phosphorylation protects Bid from cleavage by caspase-8, it allows a more profound cleavage of Bid to jBid to promote apoptosis. Furthermore, JNK can also induce TRAIL to initiate either caspase-10-associated, or tBid-activated mitochondrial-induced apoptosis [115]. Furthermore, it was shown that JNK can translocate to the mitochondria in response to TNF, where it interacts with Sh3BP5 at the mitochondrial membrane [116]. This causes a sustained JNK activation, dependent on mitochondrial ROS [117,118], which is necessary to induce apoptosis, probably via inactivating the Tyr kinase Src by DOC4-bound Tyr phosphatase SHP1 [119] or via components of the intrinsic pathway such as modulation of Bcl family members [120]. Similarly, JNK binding to isolated brain mitochondria, probably due to stimulation, has been shown to shut down mitochondrial metabolism down-regulating pyruvate dehydrogenase activity [121]. Finally, JNK was also shown to regulate apoptosis by the relatively rare autophagy-induced cell death [79]. 

#### 4.1.3. Indirect Effects through the Regulation of Transcription

In addition to the effect of the direct phosphorylation of apoptotic proteins by JNK, this kinase can also regulate the expression level of apoptotic proteins [8]. One of the key targets of JNK is the transcription factor c-Jun, which, together with other transcription factors (e.g., cFos), forms the AP-1 transcription dimer. This dimer initiates a variety of cellular processes, including apoptosis, depending on the cellular context [122]. Thus, upon apoptosis-leading activation of AP-1, the transcription factor induces the expression of regulators such as proteinases [123], TNFα, Fas-L, and Bak [124], and likely also some others that further induce the apoptotic process. Another well-known transcription factor regulated by JNK during apoptosis is p53. Indeed, it was shown that JNK phosphorylates p53 at Thr81 in the proline-rich domain (PRD), which enables the dimerization of p53 and p73 and transcription of the proapoptotic targets Puma and Bax [125]. In addition, it was shown that JNK activates p53 to induce the expression of the proapoptotic NOXA and reduce the levels of the anti-apoptotic Mcl1 that are involved in RITA (small molecule)-induced apoptosis [126]. Phosphorylated p53 can induce apoptosis by regulating the expression of death receptors, Apaf, and probably even other apoptosis-related proteins [127]. Thus, p53 is a key mediator of JNK-related apoptosis upon various stress stimuli and cell lines. Besides AP-1 and p53, other transcription factors such as FOXO1 [128], RXRα, RARβ, and AR [129] were implicated in JNK-induced apoptosis, but this requires further clarification. 

## 5. JNK-Related Induction of Apoptosis by GqPCRs

Although much is known about the regulation of the JNK cascade by many receptors and various environmental stresses, much less is known about the mode of activation and role of this cascade in GPCR signaling. GPCRs are the largest group of membranal receptors that respond to a wide variety of extracellular ligands to induce and regulate many specific physiological and pathological processes [130,131]. The transmission of GPCR signals is mediated mainly by 16 different types of heterotrimeric G proteins, which are divided into four groups: Gs, Gi, Gq, and G_12_ [132]. The components of these groups have been implicated in the activation of JNK, at least in some cell lines or tissues. Among them, the induction of JNK by Gi seems to be the most prevalent one. The mechanism of such activation may involve several distinct processes dependent on the cell line and stimulus [133]. One of two recent examples of such activation is from GnRHR-transfected COS cells, in which the activation involves EGFR, c-Src, and PI3K, [134]. The second involves κ opioid receptor-containing cells, in which JNK stimulation involves an early RAC1-dependent phase and a later arrestin-dependent phase that is mediated by RAC1 and Rho kinase [135]. Activation by Gs and G_12_ seems to be less frequent, but as in the Gi case, both of these G proteins may induce JNK activation by the small GTPase Rho [136,137]. The dissociated Gβγ by themselves have been shown to transmit intracellular signals independently of the Gα subunits [138,139]. Among other things, this complex can activate PLCβ and induce the activation of PKC [140], or PLCε and PI4P [141]. Although there are several reports that JNK can be activated specifically downstream of Gβγ (e.g., [142]), they seem to be confined to a βγ complex released from Gαi. The mechanisms involved are not well studied but may involve the induction of ROS [143] and probably also Rho [144]. In this review, we cover primarily the activation of JNK by the Gq group, which has 4 members (Gq, G_11_, G_14_, and G_15/16_) that act mainly by activating phospholipase C-β (PLCβ), which is mediated by their Gα subunit [145]. The PLCβ then produces the second messengers: inositol trisphospate, which elevates calcium levels, and diacylglycerol, which activates protein kinase C (PKC) and a few other signaling proteins. Apart from that, the Gq can also function via the receptor or βγ dimers in a PLC-independent manner to activate several other signaling events [134].

### 5.1. Activation of JNK by GqPCRs in Various Cell Types

In previous studies, our group became interested in the way in which GqPCRs transmit their signals to initiate various cellular processes. In particular, we followed the signaling by gonadotropin-releasing hormone receptor (GnRHR), which is part of a small group of receptors in which the C terminus is shorter, and therefore, their regulation is distinct from other GqPCRs [146]. Using the pituitary αT3-1 cells that express high levels of GnRHR, we found that this hormone activates JNK in a c-Src and CDC42-dependent manner [147]. Other studies showed that gonadotropin-releasing hormone analogs (GnRHa) can also cause activation of ERK [148] and p38 [149] in these cells, however, with different strengths and durations of the signals [150]. These are the main pathways that induce the GnRH effects in pituitary cells, including the induction of gonadotropins [151,152,153]. The PKC isoforms involved in the GnRHa activation of ERK were shown to include PKCβII, PKCδ, and PKCε [154]. Similarly, PKCs (particularly PKCα) seem to be involved in JNK activation in the gonadotrophs and probably other cell types (unpublished results). Interestingly, when the GnRHR was overexpressed in COS7 cells, GnRHa induced activation of ERK and JNK as well, but the mechanism that induces JNK activation was distinct from that of the αT3-1 cells, involving Gαi, Gβγ, EGFR, c-Src, and PI3K [134]. Moreover, we unexpectedly also found that, apart from the well-studied pituitary cells, GnRHR is also expressed in prostate cancer cells, where GnRH usually induces apoptosis. Thus, treatment of androgen-independent prostate cancer-derived DU145 and PC3 cell lines with GnRHa directly induces apoptosis via the JNK cascade [37]. The mechanism involved in this activation in both cells involves the activation of c-Src, either by Gi (DU145) or Gq (PC3; [155]). In turn, the c-Src induces activation of MLK3, which further transmits the signal towards the rest of the JNK cascade and induces apoptosis. Interestingly, we found that the activation of MLK3 and the rest of the JNK cascade is strongly regulated by the PI3K-AKT pathway. In both cells, the activity of AKT was reduced upon GnRHa treatment, thus releasing the AKT-induced inhibition of MLK3 to allow JNK activation. These results support the potential use of GnRHa and AKT inhibition in the treatment of advanced prostate cancer.

### 5.2. Detailed Mechanism of JNK Activation by GqPCRs/PKCs

The activation of JNK by Gq proteins via activation of PKCs was detected at the time of our study in only a few cell lines, such as gonadotrophs [147], prostate cancer cells [155], and cardiac myocyte hypertrophy [156]. Similarly, the regulation of AKT activity by PKC was rarely seen as well (e.g., Ref. [157]). We therefore performed a screen of 21 cell lines in order to determine how prevalent these signaling segments leading Gq to apoptosis might be. For this purpose, we used TPA, which bypasses the receptor/Gq level in activating PKC, and investigated the activation of JNK, reduced AKT activity, as well as the induction of apoptosis. Our results [155] show that JNK was activated in 8 out of the 21 cell lines examined, AKT was inactivated in 11, and apoptosis was induced in 7. However, all 3 signaling components were simultaneously seen in only 3 cell lines (the pituitary αT3-1, the luteinizing granulosa SVOG-4, and the prostate cancer PC3 cells), indicating that each of the signaling components may be common, but the particular wiring that was observed is relatively rare. Nonetheless, the existence of this pathway may be prevalent in cells of the reproductive system, leading to physiological induction of apoptosis in these cells as well as in some cancer cells. It is possible that GnRHR or other GqPCRs may lead to apoptosis using distinct pathways, related or not to JNK (see the above examples and refs. [158,159,160,161]). We further showed that the effects are indeed mediated by Gq. Consequently, we undertook a study aimed at elucidating the full signaling axis from Gq to apoptosis in these cells. 

**Figure 2:**
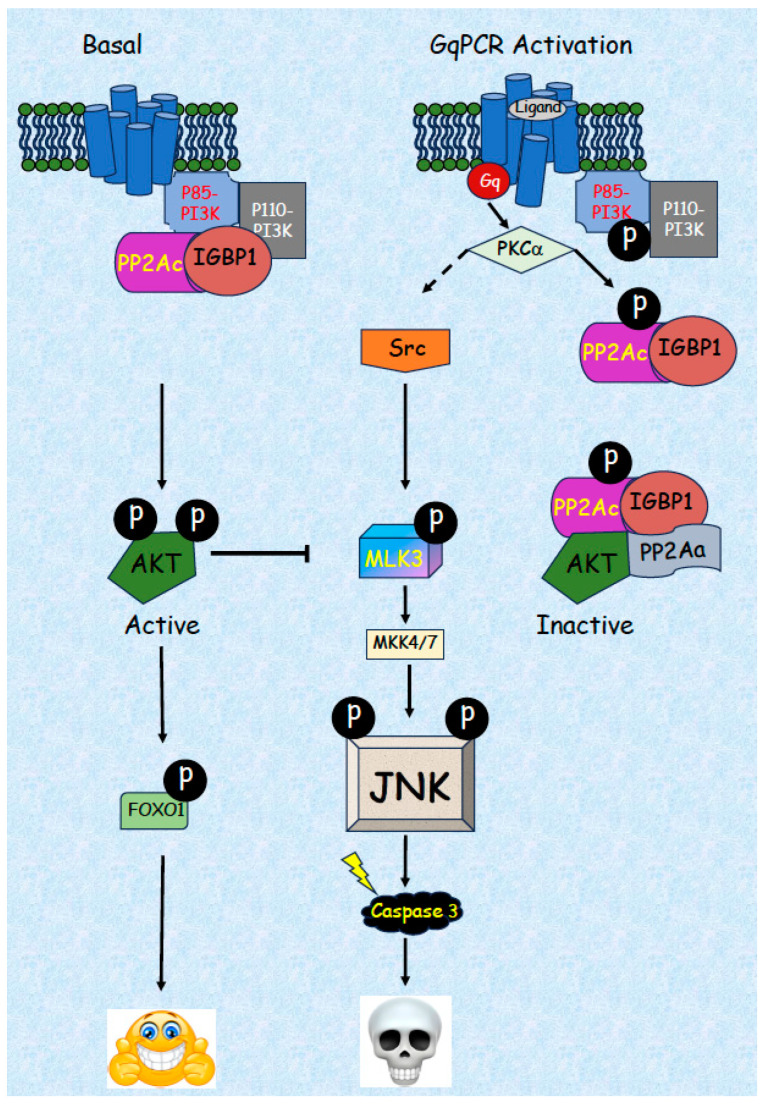
PP2A switch regulates JNK-dependent apoptosis via inhibition of PI3K and AKT activitis. In resting cells (left side) PP2Ac-IGBP1 dimer binds PI3K and maintains its activity by dephosphorylating PI3K on its autoinhibitory p85-PI3K Ser608 residue. Active PI3K activates AKT via phosphorylation, which leads to inhibition of the MLK3-MKK4/7-JNK apoptotic pathway, thus induces cell survival. Upon GqPCR activation (right panel), PKC inhibits the activator of JNK pathway Src by phosphorylation. In addition, activated PKC phosphorylates S24-PP2Ac, causing the PP2Ac-IGBP1 dimer to dissociate from PI3K (thus allowing autoinhibitory phosphorylation on p85-PI3K Ser608 residue). A heterotrimer of PP2c-IGBP1-PP2Aa is formed, binds and dephosphorylates the activatory AKT residues, rendering AKT inactive to allow alleviation of the AKT-dependent MLK3 inhibition and thus activation of the JNK pathway that leads to apoptosis.

We then studied the signaling cascades involved in the Gq-JNK-related apoptosis and found that it is mediated by two signaling branches that act downstream of PKC [155]. These two branches converge at the level of MLK3, which is the main MAP3K involved in the activation of JNK by activation of MKK7, but not MKK4 (Figure 2). The first branch is composed of c-Src activation downstream of PKC, while the other branch involves a reduction in AKT activity that alleviates its inhibitory effect on MLK3. The latter is required in order to alleviate the inhibitory AKT effect and allow the signal’s flow from c-Src to JNK. Although the first branch seems to be similar to the activation and regulation of JNK in several systems (e.g., [162]), not much is known about the mechanisms involved in the AKT-related branch. Inactivation of the JNK cascade by AKT phosphorylation of MLK3 [30] or of POSH that inhibits MLK3-JNK interaction [40] was already published in the past, and we showed that AKT inactivates MLK3 in our system by direct phosphorylation of the latter [155]. These results clearly indicate that AKT needs to be inactivated to stimulate the activation of JNK. However, the mechanism of AKT dephosphorylation and inactivation under this condition was not known. It should be emphasized that this inactivation does not involve a prior elevation of the basal activity of AKT upon stimulation that occurs by PDK1 and mTORC2 [163]. This latter inactivation is mediated in several ways, including a reduced phospholipid interaction or by lipid and protein phosphatases (PTEN, PHLPP, and PP2A; [164]). The main regulator (out of the parameters mentioned) of AKT activity shortly after its activation is PP2A, acting with the B regulatory components PR56β, PR56γ [165], or B55 [166,167], dependent on the cell’s origin. On the other hand, in the system that we studied, Gq acts on basal AKT activity without prior activation. We found that while PTEN and PHLPP are not involved, PP2A had a clear effect. Moreover, our initial results indicated that it is not regulated by the B subunits described (PR56β, PR56γ, and B55) that act to reduce stimulated AKT activity.

In order to identify the B subunit involved in the stimulated AKT inactivation, we used a SiRNA screen of all known B subunits [168] and found that it is mediated by IGBP1 (also known as α4; [169]). In resting cells, an IGBP1-PP2Ac dimer binds to PI3K, dephosphorylates the inhibitory pSer608-p85 of PI3K, and thus maintains its high basal activity. Upon stimulation, the dimer detaches from PI3K, thus allowing its autophosphorylation [170] and inactivation. In parallel, the IGBP1-PP2Ac dimer interacts with PP2Aa, and the complex binds active AKT and thus dephosphorylates and inactivates it. Although it was previously proposed that IGBP1 cannot bind PP2Ac in parallel with PP2Aa [171,172], other studies showed that such binding is possible [173,174]. Since we did observe such interactions, we propose that post-translationally modified (phosphorylated, see below) PP2Ac changes its conformation to allow the parallel binding of the two proteins that use distinct amino acids for their interaction. Taken together, we have identified a “PP2A switch”, which allows the inactivation of both PI3K and AKT, providing a unique mechanism of GPCR-stimulated dephosphorylation. However, the upstream machinery involved in the initiation of the PP2A switch still requires additional studies. Our current results suggest that PP2Ac can be phosphorylated upon stimulation by classical PKCs. Moreover, we found that this phosphorylation is involved in the PP2A switch, which ultimately induces AKT inactivation and JNK-dependent apoptosis. However, more studies are required in order to identify the phosphorylation site and how this phosphorylation induces the PP2A switch. Taken together, our results provide a novel mechanism that regulates AKT and JNK upon GqPCR stimulation in several cells. 

## 6. Conclusions

The JNK signaling cascade is a canonical MAPK pathway that transmits signals from several receptors to induce mainly stress-related cellular processes but is also known to play a role in the induction and regulation of many other processes. Dysregulation of the cascade is known to be the cause of various pathologies, including neurodegenerative and metabolic disorders, insulin resistance, inflammation, some cancers, and more. One of the main functions that is regulated by the JNK cascade is apoptosis, which is induced by several extracellular signals, mainly death receptors. Our group has shown that JNK may induce apoptosis upon stimulation of GqPCRs, a process that is detected mainly in cells of the reproductive system. In the past few years, we elucidated the mechanism involved in this induction and found that binding of ligands to some GqPCRs in several cell types activates PKCα/β, which in turn binds and phosphorylates PI3K-bound PP2Ac on its Ser24 residue. This event causes the release of the PP2Ac-IGBP1 dimer from PI3K. Since PI3K is autophosphorylated on its inhibitory Ser608 of the p85 subunit, binding of PP2A removes the phosphate and induces high basal PI3K/AKT activity. Therefore, the detachment induces autophosphorylation, which results in the inactivation of PI3K. In parallel, the released PP2Ac-IGBP1 free dimer interacts with PP2Aa, followed by binding of the formed trimer to AKT, leading to the dephosphorylation and inactivation of the latter. We termed this shift of the IGBP1-PP2Ac dimer from PI3K to AKT that causes inactivation of both kinases “a PP2A switch”, which is the principal mechanism in the induction of apoptosis upon GqPCR induction. This process is mainly regulated by PKC-induced Ser24 phosphorylation, which is necessary and sufficient to confer JNK-dependent cellular apoptosis, even in non-stimulated pituitary and prostate cancer cells. More studies are still required in order to elucidate the exact binding characteristics of all interacting components. Another important point that needs to be studied is the mechanisms that regulate the Gq-induced JNK activation described above at the upstream levels. For example, RGSs are known to suppress G protein-mediated MAPK activation [175,176], particularly JNK [177], GRKs [178], and β-arrestin/internalization [179]. Finally, our data suggest that since Gq-induced JNK activation is required to induce apoptosis in prostate cancer cells, modulating them could serve as a new strategy to combat this cancer. Further studies in these directions are recommended as well.
